# Oral modified release morphine for breathlessness in chronic heart failure: a randomized placebo‐controlled trial

**DOI:** 10.1002/ehf2.12498

**Published:** 2019-08-06

**Authors:** Miriam J. Johnson, Sarah Cockayne, David C. Currow, Kerry Bell, Kate Hicks, Caroline Fairhurst, Rhian Gabe, David Torgerson, Laura Jefferson, Stephen Oxberry, Justin Ghosh, Karen J. Hogg, Jeremy Murphy, Victoria Allgar, John G.F. Cleland, Andrew L. Clark

**Affiliations:** ^1^ Wolfson Palliative Care Research Centre University of Hull Hull HU6 7RX UK; ^2^ York Trials Unit University of York York UK; ^3^ IMPACCT, Faculty of Health University of Technology Sydney Ultimo NSW Australia; ^4^ Hull York Medical School and York Trials Unit University of York York UK; ^5^ Calderdale & Huddersfield Foundation Trust Huddersfield Royal Infirmary Huddersfield UK; ^6^ Department of Cardiology Scarborough Hospital Scarborough UK; ^7^ Department of Cardiology Glasgow Royal Infirmary, University of Glasgow Glasgow UK; ^8^ Department of Cardiology Darlington Memorial Hospital Darlington UK; ^9^ Hull York Medical School and Department of Health Sciences University of York York UK; ^10^ Robertson Centre for Biostatistics & Clinical Trials, Institute of Health & Well‐being University of Glasgow Glasgow UK; ^11^ Hull York Medical School Castle Hill Hospital Cottingham UK

**Keywords:** Heart failure, Randomized controlled trial, Morphine, Breathlessness, Dyspnoea

## Abstract

**Aims:**

Morphine is shown to relieve chronic breathlessness in chronic obstructive pulmonary disease. There are no definitive data in people with heart failure. We aimed to determine the effectiveness and cost‐effectiveness of 12 weeks morphine therapy for the relief of chronic breathlessness in people with chronic heart failure compared with placebo.

**Methods and results:**

Parallel group, double‐blind, randomized, placebo‐controlled, phase III trial of 20 mg daily oral modified release morphine was conducted in 13 sites in England and Scotland: hospital/community cardiology or palliative care outpatients. The primary analysis compared between‐group numerical rating scale average breathlessness/24 hours at week 4 using a covariance pattern linear mixed model. Secondary outcomes included treatment‐emergent harms (worse or new). The trial closed early due to slow recruitment, randomizing 45 participants [average age 72 (range 39–89) years; 84% men; 98% New York Heart Association class III]. For the primary analysis, the adjusted mean difference was 0.26 (95% confidence interval, −0.86 to 1.37) in favour of placebo. All other breathlessness measures improved in both groups (week 4 change‐from‐baseline) but by more in those assigned to morphine. Neither group was excessively drowsy at baseline or week 4. There were no between‐group differences in quality of life (Kansas) or cognition (Montreal) at any time point. There was no exercise‐related desaturation and no change between baseline and week 4 in either group. There was no change in vital signs at week 4. The natriuretic peptide measures fell in both groups but by more in the morphine group [morphine 2169 (1092, 3851) pg/mL vs. placebo 2851 (1694, 5437)] pg/mL. There was no excess serious adverse events in the morphine group. Treatment‐emergent harms during the first week were more common in the morphine group; all apart from 1 were ≤ grade 2.

**Conclusions:**

We could not answer our primary objectives due to inadequate power. However, we provide novel placebo‐controlled medium‐term benefit and safety data useful for clinical practice and future trial design. Morphine should only be prescribed in this population when other measures are unhelpful and with early management of side effects.

## Introduction

Although modern medical therapy is successful in improving morbidity and mortality in patients with chronic heart failure, for some, breathlessness persists^1^ despite optimal pharmacological therapy. Persisting breathlessness is associated with poorer physical and mental quality of life,^2^ impaired activities of daily living,^3^ increased unplanned hospital attendance^4^ and admissions,^5^ and higher mortality.^3^ Although non‐pharmacological and pharmacological interventions can reduce its impact, its importance to patients is often neglected in guidelines and clinical trials.^6^, ^7^, ^8^

The perception of breathlessness is processed in brain areas^9^ rich in opioid receptors.^10^ Endogenous opioids reduce breathlessness, whereas the opioid antagonist, naloxone, increases exertion‐induced breathlessness by blocking the effects of endogenous opioids on the brain.^11^, ^12^ In people with chronic breathlessness due to a range of causes, but mainly chronic obstructive pulmonary disease, regular, low dose, modified release morphine is safe and effective in the short term (7 days).^7^, ^13^ However, the evidence is less clear in people with chronic heart failure.^14^, ^15^ Preliminary data suggest that people with chronic heart failure may benefit from morphine given for 3 months.^16^ Despite the lack of definitive data, morphine is used in clinical practice although there is wide variation in willingness to prescribe, dosing, and quality of monitoring. Potential problems are (i) patients may be denied a helpful medication (due to unfounded fears about harms^17^ and addiction);^18^ (ii) they may have a poorly monitored, suboptimal regime; and (iii) there may be no net benefit in the longer term (although there is no evidence of tachyphylaxis to date).

We therefore designed BreatheMOR‐HF to determine whether morphine therapy given for up to 12 weeks is superior to placebo for the relief of chronic breathlessness in ambulatory patients with chronic heart failure who remained symptomatic despite guideline‐recommended medical therapy. The trial closed early due to poor recruitment but collected important medium‐term placebo‐controlled data especially on toxicity and safety, which we report here.

## Methods

### Trial design

BreatheMOR‐HF was a 12 week, parallel group, double‐blind, randomized, placebo‐controlled, fixed dosed, multi‐site, phase III trial of 20 mg daily oral modified release morphine measuring breathlessness intensity in ambulatory patients with symptomatic chronic heart failure.

### Participants and setting

Patients from 13 centres in England and Scotland, attending hospital/community cardiology or palliative care clinics or hospices, were screened by research nurses in conjunction with the patients' usual clinical team. Eligible participants (i) were aged ≥18 years; (ii) had New York Heart Association (NYHA) class III/IV symptoms; (iii) had either left ventricular systolic dysfunction defined as left ventricular ejection fraction <40% or left ventricular ejection fraction >40% and left ventricular hypertrophy, left atrial dilation or abnormal diastolic function; (iv) had N‐terminal‐pro‐B‐type natriuretic peptide ≥1000 pg/mL or B‐type natriuretic peptide ≥250 pg/mL within the last 3 months; (v) were on a guideline‐recommended medical treatment for chronic heart failure and unchanged for ≥2 weeks; (vi) had a glomerular filtration rate ≥30 mL/min(/1.73m^2^) within 2 weeks; and (vii) scored ≥grade 2 on the modified Medical Research Council (mMRC) breathlessness scale.

Optimal medical management for people with reduced left ventricular function was defined as a maximally tolerated dose of an inhibitor of the renin‐angiotensin system and a beta‐adrenoceptor antagonist and a mineralocorticoid receptor antagonist. People with preserved left ventricular function were required only to receive diuretics and treatment for ventricular rate control for atrial fibrillation. Patients unable to provide written informed consent or complete study questionnaires, had co‐existing relevant neoplasia, had used opioids regularly within the last month at a daily dose ≥ study dose, or had a documented contra‐indication to morphine were excluded.

### Randomization

Random allocations (1:1; stratified block randomization by centre; randomly permuted block sizes of 2 and 4; investigators blinded to block size) were centrally generated by an online secure service (sealed envelope™) following eligibility data entry of consented participants by a site researcher.

### Approvals

The protocol, amendments, and trial documentation were approved by the North West‐Liverpool Central Research Ethics Committee (ref. 14/NW/0277; 7 January 2014). Medicines and Healthcare products Regulatory Agency approval was received (9 November 2014). NHS site approvals were obtained, and the trial was registered (ISRCTN41349358) prior to recruitment.

### Intervention and comparator

Participants were allocated to capsules of 10 mg modified release morphine [MST® CONTINUS® (https://www.medicines.org.uk/emc/product/7666/smpc)] or placebo that were identical in appearance, taste, and smell. Capsules were to be taken orally twice daily.

### Blinding

Participants, research team members, and clinicians were blind to treatment allocation. Site pharmacists received the capsules unblinded with a tear‐off strip to allow blinding at the time of dispensing. To prevent unblinding due to constipation, a laxative (100 mg docusate) capsule was given twice daily to patients assigned to morphine and an identical placebo to those assigned to the placebo‐control group.

### Procedures

Participants' demographic and clinical details were recorded at baseline prior to randomization. Serum urea, electrolytes, and creatinine were measured within 2 weeks of randomization. Renal clearance was assessed using estimated glomerular filtration rate or calculated using the Cockroft and Gault method.^19^ The Charlson Comorbidity Score^20^ and modified Medical Research Council breathlessness scale^21^ were also recorded at baseline.

Outcome data were collected at days 2, 4, and 7 and weeks 2, 3, 4, 8, and 12 after randomization during home or clinic visits or by telephone (depending upon the outcomes and patient preference).

The primary endpoint was measured at week 4. Capsules were dispensed at baseline and at 4 and 8 weeks. Each time, 56 morphine/placebo and 56 placebo/docusate capsules were dispensed. Participants were advised not to drive during the first week and asked to return unused capsules for compliance reconciliation.

At the end of 12 weeks, participants could choose whether to take open‐label morphine following the same regimen as the trial but prescribed and monitored by their usual‐care clinician. The trial was closed early, due to slow recruitment, in May 2018; the last participant completed follow‐up in August 2018.

### Outcomes

The primary outcome measure was the average numerical rating scale breathlessness intensity score over the previous 24 h^22^ assessed at 4 weeks. *Table* 1 details primary and secondary outcomes.

**Table 1:** Overview of primary and secondary outcomes

Primary outcome	Average breathlessness over the previous 24 h [baseline, D2, D4, D7, W2, W3, W4 (primary time point), W8, and W12]
	○ 0–10 (11 points) NRS^22^
	○ 0 = none to 10 = worst imaginable
Other breathlessness assessments	Intensity of worst breathlessness over the previous 24 h; distress due to breathlessness over the previous 24 h; unpleasantness of breathlessness over the previous 24 h (baseline, D2, D4, D7, W2, W3, W4, W8, and W12)
	○ 0–10 (11 points) NRS^22^
	○ 0 = none to 10 = worst imaginable
	Global impression of change (W4)^23^
	○ Subjective measure of response to treatment
	○ Participants asked if their breathlessness has changed and by how much using a verbal rating scale
Related symptoms	Average pain over previous 24 h (baseline, D2, D4, D7, W2, W3, W4, W8, and W12)
	○ 0–10 (11 point) NRS^24^
	○ 0 = no pain to 10 = worst imaginable pain
	Epworth Sleepiness Scale (ESS; baseline and W4)^25^
	○ Screening tool for sleep‐disordered breathing
	○ Specifically distinguishes reports of daytime dozing behaviour from fatigue and drowsiness/sleepiness
	○ Scores between 0 and 24
	○ Higher scores indicate excessive sleepiness (11–12 mild; 13–16 moderate; >16 severe)
Functional and performance status	6 min walk test (6MWT; baseline and W4)^26^
	○ Recorded distance walked in metres and O_2_ saturation at rest and post‐test
	Physical activity monitoring (activPAL™ step count; baseline and W4)^27^
	○ activPAL™ worn for 7 days at baseline prior to randomization and for 7 days prior to week 4
	○ Discriminates between sedentary, upright, and stepping activities
	○ Average daily step count documented
	Montreal Cognitive Assessment (MoCA; baseline and W4. Shortened telephone‐based MoCA administered at D4 and D7)^28^
	○ 30‐item questionnaire assessing cognitive function
	○ Scores between 0 and 30; ≥26 implies no cognitive impairment (telephone version scored 0 to 16)
	○ Items that could be administered by phone assessed on Days 4 and 7
	New York Heart Association class (NYHA; baseline, W4, and W12)^29^
	○ Four classes based on symptoms (I, II, III, and IV)
	○ Class IV denotes worst symptom status
	Australia‐modified Karnofsky Performance Status (AKPS; baseline, W4, and W12)^30^
	○ Validated variant of Karnofsky Performance Status
	○ Scored 0 to 100 in increments of 10 assigned to participants based on ability to perform activities of daily living; higher scores imply better function
Quality of life	Kansas City Cardiomyopathy Questionnaire‐short form (KCCQ‐12; baseline, W4, and W12)^31^
	○ 12‐item, self‐administered instrument quantifying physical function, symptoms (frequency, severity, and recent change), social function, self‐efficacy and knowledge, and quality of life
	○ Combined single, overall summary score between 0 and 100
	○ Higher scores indicate better functioning, fewer symptoms, and better disease‐specific quality of life
Harms^a^	Opioid‐relevant symptoms during each assessment using criteria established by the National Cancer Institute (version 4.03) and graded accordingly (baseline, D2, D4, D7, W2, W3, W4, W8, and W12)^32^
	○ Constipation
	○ Confusion
	○ Nausea
	○ Vomiting
	○ Memory impairment
	○ Cognitive impairment
	Karolinska Sleepiness Scale (KSS; baseline, D2, D4, D7, W2, W3, and W4)^33^
	○ 9‐point Likert scale of the patient's level of drowsiness (1 = very alert to 9 = very sleepy)
Health economic assessment	EuroQoL EQ‐5D‐5L (baseline, W4, W8, and W12)^34^
	○ Self‐administered, validated measure of health status
	○ Five dimensions: mobility, self‐care, usual activities, pain/discomfort, and anxiety/depression
	○ Five levels [Level 1 = no problems, Level 2 = slight problems, Level 3 = moderate problems, Level 4 = severe problems, Level 5 = unable (or extreme)] and a visual analogue self‐rating scale
	Health service use (baseline, W4, W8, and W12)
	○ Participants recall specified service use over the past 4 weeks
Clinical assessments	Standard examination (baseline and W4)
	○ Resting pulse rate and blood pressure
	○ Resting respiratory rate
	○ Pulse oximetry
	N‐terminal proBNP (B‐type natriuretic peptide) measurement (baseline and W4)
	○ For sites with access to this test as part of clinical practice
	Dose of ‘as required' immediate release opioid for breathlessness (W4 and W12)
	○ Patient diary: if, when, and the dose of any ‘as required' dose of immediate release opioid solution taken for breathlessness

a Known opioid‐related adverse events were measured at baseline and during follow‐up.

### Sample size

Based on our previous data,^15^ a 1 point difference on the breathlessness scale was chosen to demonstrate a minimum clinically important difference. In order to detect this difference between the groups at 4 weeks with 90% power at 5% significance (and assuming a standard deviation of 2.55, giving a medium effect size of 0.4), 138 patients were required in each group. Allowing for 20% attrition, we needed 346 patients (173 to each group).

### Statistical analysis

Analyses were conducted in Stata v13 (StataCorp. 2013. Stata Statistical Software: Release 13. College Station, TX: StataCorp LP) on an intention‐to‐treat basis. Statistical tests were two‐sided at 5% significance level. Baseline data are summarized overall and by trial arm both by randomization and separately for participants providing data to the primary endpoint. No statistical comparisons between treatment groups were undertaken on baseline data.

The primary analysis compared the NRS average breathlessness at week 4 between the morphine and placebo groups using a covariance pattern linear mixed model. The outcomes were numerical rating scale at each post‐randomization time point (≤week 12), nested within patients. Scores at baseline, trial arm, time point, and a time‐by‐trial arm interaction were included as fixed‐effects with participant and site as random‐effects. An independent covariance structure for the repeated measurements was used as this provided the smallest Akaike's information criterion.^35^

The adjusted mean difference, with its associated 95% confidence interval and *P* value, between the two groups for the week 4 time point was extracted from the model.

The secondary outcomes of the Australian‐modified Karnofsky Performance Scale, Kansas City Cardiomyopathy Questionnaire‐12, and Karolinska Sleepiness Scale were similarly analysed. The Epworth Sleep Scale, Montreal Cognitive Assessment, 6 min walk test, and activPAL™ were analysed using mixed‐effect linear regression to compare the scores at week 4 adjusting for baseline score and site as a random effect (i.e. repeated measures per participant not required to be included).

Data on study drug use are described in Supporting Information, *Table* *S5*, with adverse event data presented by trial arm in Supporting Information, *Tables* *S4A* and *S4B* and on harms in Supporting Information, *Tables* *S5A* and *S5B*.

A full cost‐effectiveness analysis was originally planned; however, as the study is underpowered, EQ‐5D‐5L and health resource use data are summarized descriptively (Supporting Information, *Tables* *S4A* and *S4B*).

This study report uses the CONSORT framework for reporting randomized clinical trials.^36^

## Results

Thirteen sites opened to recruitment and seven randomized at least one participant. The first participant was randomized in June 2016 and recruitment closed in May 2017; by which time, 45 patients had been recruited and randomized (21 to morphine and 24 to placebo).

**Figure 1:**
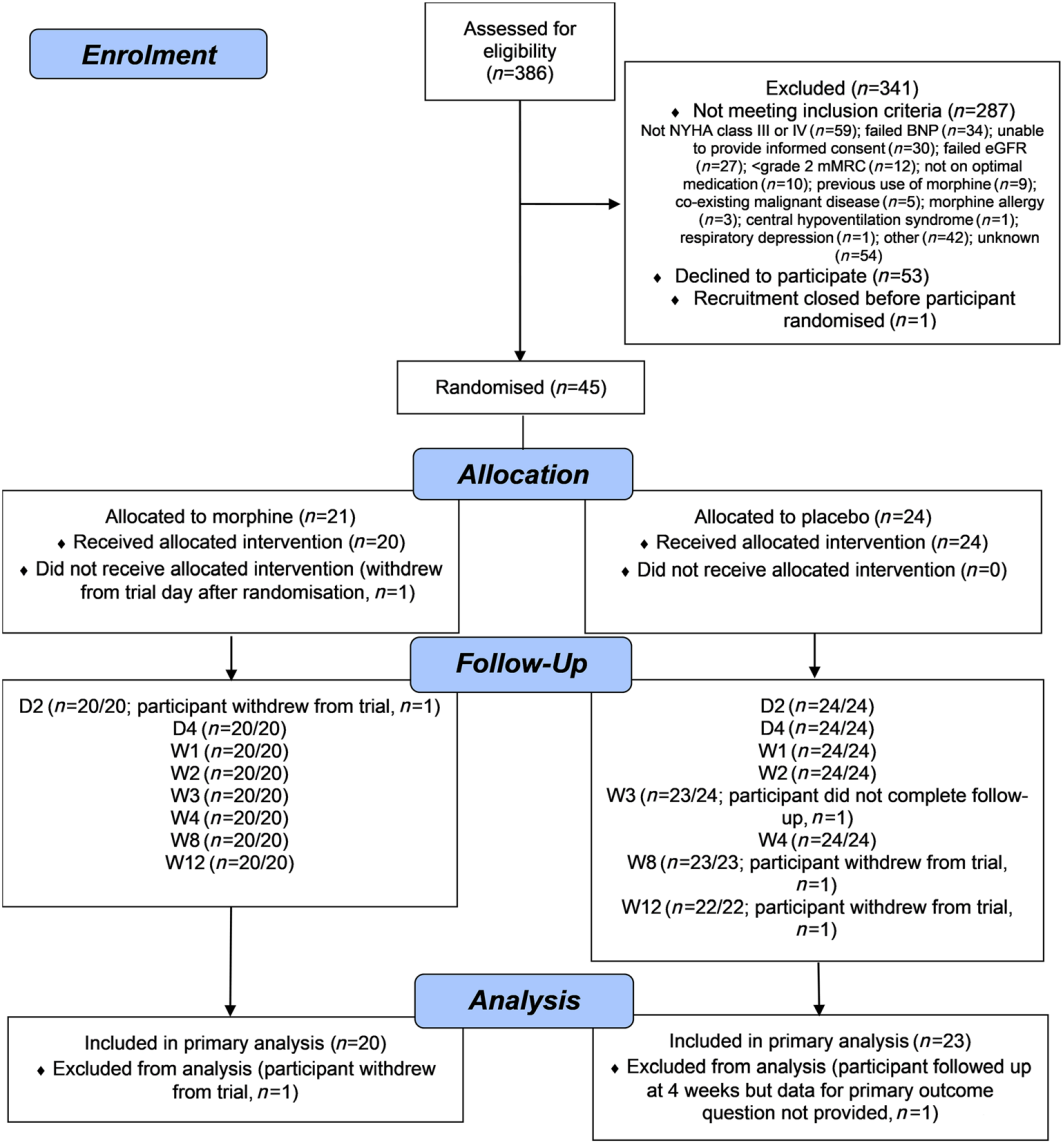
CONSORT flow diagram of participant through the BreatheMOR‐HF trial.

Altogether, 386 patients were screened between December 2015 and May 2017 (median 27 per site, range 0–55), of whom 287 (74%) were ineligible, 53 (14%) declined, and one (0.3%) was eligible but the trial closed prior to their randomization. The most common reasons for ineligibility were the absence of NYHA functional class III or IV (*n* = 59) and natriuretic peptide plasma concentrations below the inclusion criteria (*n* = 34) (*Figure* *1*).

The average age of randomized participants was 72 years (range 39–89), and 84% were men (*Table* 2). All but one had NYHA class III symptoms, and 78% had mMRC breathlessness grade 3 or 4. Baseline characteristics were generally well balanced, but those assigned to placebo had more severe breathlessness on the mMRC scale (*Table* 2).

**Table 2:** Baseline characteristics by randomized group, as randomized and as included in the primary outcome analysis

	As randomized		As analysed	
Characteristic^a^	Morphine (*n* = 21)	Placebo (*n* = 24)	Morphine (*n* = 20)	Placebo (*n* = 23)
Sex				
Male	18 (85.7)	20 (83.3)	17 (85.0)	20 (87.0)
Female	3 (14.3)	4 (16.7)	3 (15.0)	3 (13.0)
Age, years	74.4 (6.0)	70.1 (14.0)	74.1 (6.0)	71.5 (12.6)
Ethnicity				
White	21 (100.0)	24 (100.0)	21 (100.0)	24 (100.0)
NYHA class				
III	20 (95.2)	24 (100.0)	19 (95.0)	23 (100.0)
IV	1 (4.8)	0 (0.0)	1 (5.0)	0 (0.0)
Resting pulse rate (per min) (radial)	77.0 (24.0)	77.0 (11.2)	76.8 (24.6)	77.1 (11.4)
Resting systolic blood pressure, mmHg	119.8 (24.2)	116.1 (14.5)	121.3 (23.8)	116.4 (14.8)
Resting diastolic blood pressure, mmHg	69.4 (12.3)	68.0 (11.6)	70.2 (12.1)	68.7 (11.2)
Resting respiratory rate (per min)	17.9 (6.8)	15.6 (4.4)	18.2 (6.8)	15.4 (4.4)
Pulse oximetry, %	97.1 (2.1)	96.7 (1.6)	97.1 (2.1)	96.7 (1.7)
mMRC grade^b^				
0	0 (0.0)	0 (0.0)	0 (0.0)	0 (0.0)
1	0 (0.0)	0 (0.0)	0 (0.0)	0 (0.0)
2	7 (33.3)	3 (12.5)	7 (35.0)	3 (13.0)
3	11 (52.4)	21 (87.5)	10 (50.0)	21 (87.0)
4	3 (14.3)	0 (0.0)	3 (15.0)	0 (0.0)
eGFR, mL/min	53.0 (18.2)	62.2 (21.4)	53.9 (18.2)	61.8 (21.8)
NTproBNP, pg/mL^c^	*N* = 20, 2963 (1883, 4743)	*N* = 22, 2587 (1436, 4636)	*N* = 19, 2843 (1860, 4230)	*N* = 21, 2646 (1761, 4636)
BNP, pg/mL	*N* = 1, 528 (−)	*N* = 2, 844 (−)	*N* = 1, 528 (−)	*N* = 2, 844 (−)
Charlson Comorbidity Index	6.7 (1.4)	6.2 (2.3)	6.7 (1.5)	6.4 (2.0)

a Continuous data are presented as mean (SD) and categorical data as *n* (%).

b 0 = not troubled by breathlessness except on strenuous exercise; 1 = short of breath when hurrying or walking up a slight hill; 2 = walks slower than contemporaries on the level because of breathlessness or has to stop for breath when walking at own pace; 3 = stops for breath after about 100 m or after a few minutes on the level; 4 = too breathless to leave the house or breathless when dressing or undressing.

c NTproBNP conducted by certain sites only; data are presented as median and interquartile range.

**Figure 2:**
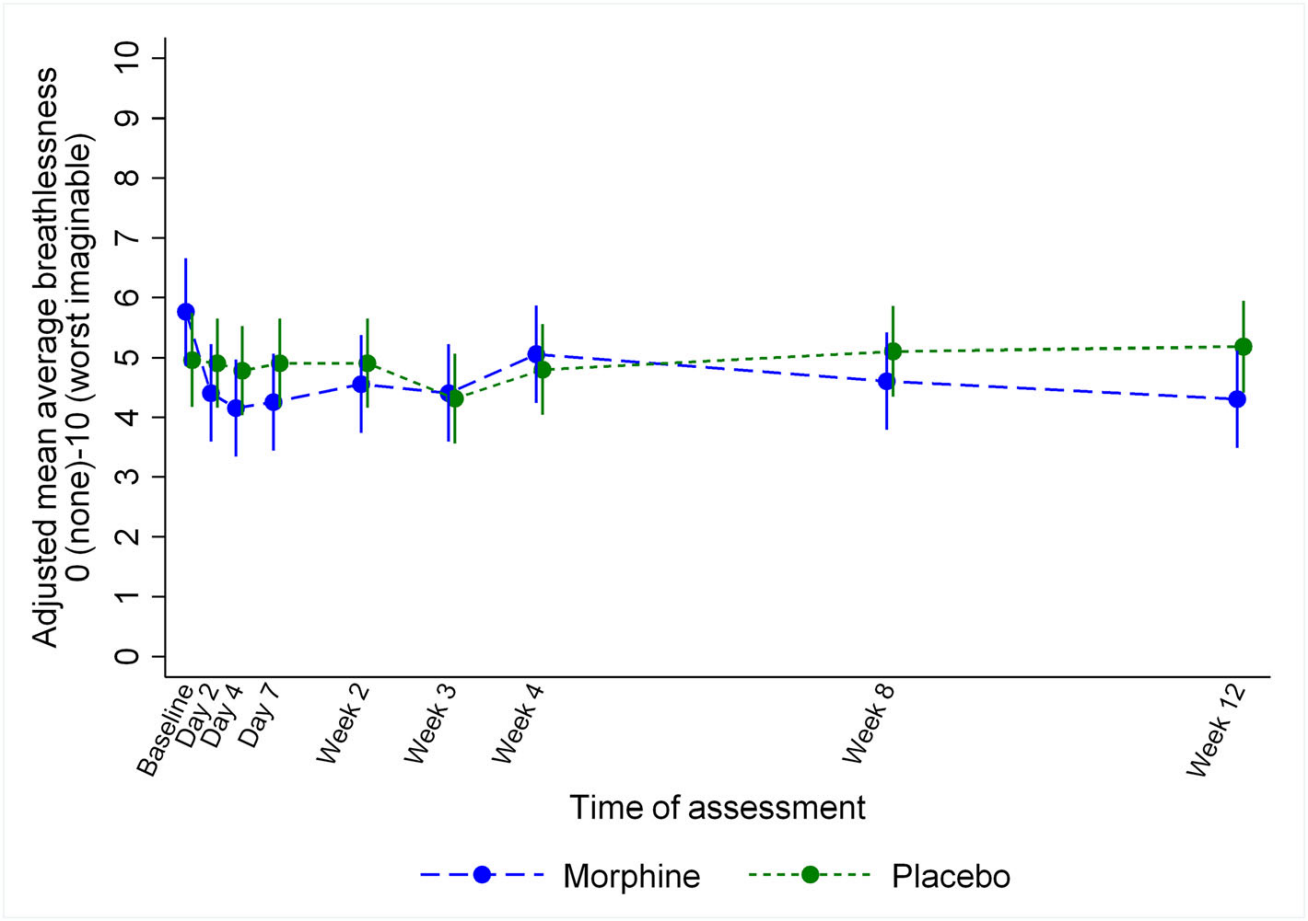
Mean average breathlessness by randomized group and time point as measured on a numerical rating scale from 0 (no breathlessness) to 10 (worst imaginable breathlessness) adjusted for baseline NRS.

For the primary endpoint, the raw mean (standard deviation) scores were 5.3 (2.3) for those assigned to morphine (*n* = 20) and 4.6 (2.4) for those assigned to placebo (*n* = 23) (*Table* 3). The adjusted mean difference was 0.26 (95% confidence interval, −0.86 to 1.37, *P* = 0.65) in favour of the placebo group (*Figure* *2*). No adjusted mean difference of 1 point or more (clinically important difference) was observed at 4 weeks between the groups for any NRS item.

**Table 3:** Raw NRS summary scores for breathlessness by randomized group and time point, with adjusted mean difference between the groups at primary time point of 4 weeks

NRS [0 (best) to 10 (worst)] *N*, Mean (SD)	Time point	Morphine (*n* = 21)	Placebo (*n* = 24)	Total (*n* = 45)	Adjusted mean difference at W4 (95% CI), *P* value
How bad has your breathlessness felt on average over the past 24 h?	Baseline	21, 5.8 (2.0)	24, 5.0 (1.9)	45, 5.3 (1.9)	0.26 (−0.86 to 1.37), *P* = 0.65
	D2	20, 4.7 (2.1)	24, 4.7 (1.6)	44, 4.7 (1.8)	
	D4	20, 4.4 (2.1)	24, 4.5 (1.7)	44, 4.5 (1.9)	
	D7	20, 4.6 (2.5)	24, 4.7 (1.7)	44, 4.6 (2.1)	
	W2	20, 4.8 (2.4)	24, 4.7 (2.0)	44, 4.8 (2.1)	
	W3	20, 4.7 (2.4)	23, 4.0 (2.2)	43, 4.3 (2.3)	
	W4	20, 5.3 (2.3)	23, 4.6 (2.4)	43, 4.9 (2.4)	
	W8	20, 4.9 (2.4)	23, 4.9 (2.1)	43, 4.9 (2.2)	
	W12	20, 4.6 (2.5)	22, 5.0 (2.2)	42, 4.8 (2.4)	
How bad has your breathlessness felt at its worst over the past 24 h?	Baseline	21, 7.2 (2.4)	24, 6.2 (1.9)	45, 6.7 (2.2)	0.15 (−1.13 to 1.44), *P* = 0.82
	D2	20, 5.2 (2.1)	24, 5.2 (2.2)	44, 5.2 (2.1)	
	D4	20, 4.5 (2.5)	24, 5.1 (2.5)	44, 4.8 (2.5)	
	D7	20, 5.2 (2.8)	24, 5.3 (2.3)	44, 5.3 (2.5)	
	W2	20, 5.3 (2.5)	24, 5.1 (2.0)	44, 5.2 (2.2)	
	W3	20, 5.0 (2.3)	23, 4.4 (2.5)	43, 4.7 (2.4)	
	W4	20, 5.9 (2.5)	23, 5.3 (2.6)	43, 5.6 (2.5)	
	W8	20, 6.0 (2.8)	23, 5.3 (2.5)	43, 5.6 (2.6)	
	W12	20, 5.1 (2.8)	22, 5.5 (2.0)	42, 5.3 (2.4)	
How unpleasant has your breathlessness been on average over the past 24 h?	Baseline	21, 5.6 (2.4)	24, 4.5 (2.0)	45, 5.0 (2.2)	−0.15 (−1.48 to 1.17), *P* = 0.82
	D2	20, 4.3 (2.2)	24, 4.0 (1.8)	44, 4.1 (2.0)	
	D4	20, 4.0 (2.2)	24, 3.8 (2.1)	44, 3.9 (2.1)	
	D7	20, 4.4 (2.8)	24, 3.8 (2.1)	44, 4.1 (2.5)	
	W2	20, 4.3 (2.7)	24, 4.4 (2.2)	44, 4.4 (2.4)	
	W3	19, 3.8 (2.1)	23, 2.9 (2.2)	42, 3.3 (2.2)	
	W4	20, 4.7 (2.8)	23, 4.3 (2.1)	43, 4.4 (2.4)	
	W8	20, 4.3 (2.6)	23, 4.1 (2.5)	43, 4.2 (2.6)	
	W12	20, 4.3 (3.0)	22, 4.3 (2.6)	42, 4.3 (2.7)	
How much distress has your breathlessness caused you on average over the past 24 h?	Baseline	21, 5.7 (2.4)	24, 4.1 (2.3)	45, 4.8 (2.5)	−0.55 (−1.99 to 0.88), *P* = 0.45
	D2	20, 3.3 (2.5)	24, 3.3 (2.1)	44, 3.3 (2.2)	
	D4	20, 2.7 (2.5)	24, 3.1 (2.7)	44, 2.9 (2.6)	
	D7	20, 3.5 (3.0)	24, 3.3 (2.3)	44, 3.4 (2.6)	
	W2	20, 3.8 (3.2)	24, 3.1 (2.6)	44, 3.4 (2.9)	
	W3	20, 3.3 (2.7)	22, 2.8 (2.4)	42, 3.0 (2.5)	
	W4	20, 4.2 (3.3)	23, 3.8 (2.6)	43, 4.0 (2.9)	
	W8	20, 4.2 (3.1)	23, 3.6 (2.6)	43, 3.8 (2.8)	
	W12	20, 3.8 (2.9)	22, 4.0 (2.4)	42, 3.9 (2.6)	
How much pain have you had on average over the past 24 h?	Baseline	21, 1.9 (3.1)	24, 1.2 (2.1)	45, 1.5 (2.6)	−0.05 (−1.29 to 1.20), *P* = 0.94
	D2	20, 1.3 (2.4)	24, 1.3 (2.0)	44, 1.3 (2.1)	
	D4	20, 1.3 (2.5)	24, 0.9 (1.7)	44, 1.0 (2.1)	
	D7	18, 0.8 (1.9)	24, 0.7 (1.6)	42, 0.8 (1.7)	
	W2	20, 1.3 (2.5)	24, 0.8 (1.4)	44, 1.0 (2.0)	
	W3	20, 0.9 (1.9)	23, 1.0 (1.6)	43, 0.9 (1.7)	
	W4	20, 1.5 (2.8)	23, 1.1 (1.9)	43, 1.3 (2.3)	
	W8	19, 1.9 (3.4)	23, 1.8 (3.0)	42, 1.8 (3.1)	
	W12	20, 2.0 (3.3)	22, 0.9 (2.0)	42, 1.4 (2.8)	

From baseline to week 4, breathlessness measures, notably unpleasantness of, and distress due to, breathlessness improved in both groups (*Table* 3). The improvement was greater in those assigned to morphine compared with placebo in all but average intensity. All breathlessness scores increased further during subsequent weeks in those assigned to morphine but not in those assigned to placebo. Subjective global impression of change ratings are presented in Supporting Information, *Table* *S1*.

The median Australian‐modified Karnofsky Performance Status was 70 for both groups across all time points (*Table* 4; secondary outcomes). Neither group was excessively sleepy or drowsy at baseline or week 4. There were no between‐group differences in the quality of life (Kansas) or cognition (Montreal) at any time point. At week 4, there was a raw mean difference of 1113 steps per day favouring the placebo group (activPAL™) but of 7.4 m in the walk test favouring the morphine group. There was no exercise‐related desaturation and no change between baseline and week 4 in either group. There was no change in vital signs at week 4. The natriuretic peptide measures fell in both groups but by more in the morphine group (Supporting Information, *Table* *S2*).

**Table 4:** Other secondary outcome scores by randomized group and time point

Outcome	Morphine (*n* = 21)		Placebo (*n* = 24)		Total (*n* = 45)		Adjusted mean difference at W4 (95% CI), *P* value
Australian‐modified Karnofsky Performance Status [10 (comatose) to 100 (normal)]							
Baseline	21	70 (60, 80)	24	70 (60, 70)	45	70 (60, 70)	−2.1 (−7.0 to 2.8), *P* = 0.40
Week 4	20	70 (60, 75)	22	70 (70, 70)	42	70 (70, 70)	
Week 12	22	70 (60, 80)	20	70 (60, 80)	42	70 (60, 80)	
Cardiomyopathy Questionnaire (Kansas City) [1 (extremely limited) to 100 (not limited)]							
Baseline	21	36.6 (14.7)	24	40.2 (11.9)	45	38.5 (13.2)	−2.7 (−9.7 to 4.3), *P* = 0.44
Week 4	20	37.2 (16.0)	22	44.1 (12.9)	42	40.8 (14.7)	
Week 12	20	42.2 (22.0)	22	42.3 (17.7)	42	42.2 (19.6)	
Epworth Sleepiness Scale (0–24; higher score = greater sleepiness)							
Baseline	21	9.6 (4.1)	24	9.5 (4.8)	45	9.6 (4.5)	1.3 (−0.8 to 3.5), *P* = 0.23
Week 4	20	10.6 (5.2)	22	9.4 (4.3)	42	10.0 (4.8)	
Karolinska Sleepiness Scale (1 = very alert to 9 = very sleepy)							
Baseline	21	3.0 (1.5)	24	3.3 (1.6)	45	3.2 (1.5)	0.3 (−0.5 to 1.2), *P* = 0.45
Day 2	20	3.8 (1.7)	24	3.4 (1.2)	44	3.6 (1.4)	
Day 4	20	3.8 (1.9)	24	3.8 (1.9)	44	3.8 (1.8)	
Day 7	20	4.6 (2.5)	24	3.5 (1.7)	44	4.0 (2.2)	
Week 2	20	3.2 (1.4)	24	3.5 (1.9)	44	3.4 (1.7)	
Week 3	20	3.1 (1.4)	23	3.2 (1.8)	43	3.1 (1.6)	
Week 4	20	3.3 (1.5)	23	3.0 (1.6)	43	3.2 (1.5)	
Montreal Cognitive Assessment [0–30 (0–16 phone version); lower scores = greater cognitive impairment]							
Baseline	21	25.1 (1.9)	24	25.4 (3.1)	45	25.2 (2.6)	−0.5 (−2.2 to 1.1), *P* = 0.53
Day 4 (phone version)	19	14.1 (1.3)	21	14.3 (1.9)	40	14.2 (1.6)	
Day 7 (phone version)	18	14.2 (1.1)	23	14.7 (1.3)	41	14.5 (1.2)	
Week 4	20	26.2 (3.3)	21	26.8 (2.3)	41	26.5 (2.8)	
6 min walk test [distance walked (m)]							
Baseline	18	153 (105, 273)	24	179 (133, 255)	42	160 (120, 270)	18.7 (−48.8 to 86.3), *P* = 0.59
Week 4	13	169 (120, 250)	17	165 (90, 270)	30	167 (104, 270)	
O_2_ saturation at rest (%)							
Baseline	18	97 (96, 99)	24	97 (95, 98)	42	97 (96, 98)	−0.7 (−1.8 to 0.4), *P* = 0.23
Week 4	13	96 (95, 98)	16	97 (96, 98)	29	97 (95, 98)	
O_2_ saturation at end (%)							
Baseline	18	98 (97, 98)	24	97 (96, 99)	42	98 (97, 99)	−0.2 (−1.3 to 0.9), *P* = 0.74
Week 4	13	97 (96, 98)	16	97 (96, 99)	29	97 (96, 98)	
activPAL™ (average steps per day)							
Baseline	20	2503 (976, 3700)	22	2207 (473, 3183)	42	2315 (589, 3445)	−728.2 (−1438.5 to −17.8), *P* = 0.05
Week 4	19	1943 (361, 2975)	17	2717 (1744, 3143)	36	2259 (1061, 3063)	

Data are *N*, mean (SD) or *N*, median (IQR).

Adherence is summarized in Supporting Information, *Table* *S3*. All but one participant took at least one trial capsule. One participant assigned to morphine withdrew the day after randomization. Three participants withdrew fully from the trial (i.e. from treatment and follow‐up, *Figure* *1*) and 16 participants [11 (52.4%) assigned to morphine and five (20.8%) assigned to placebo] formally withdrew from treatment before the 12 week assessment [median time to treatment withdrawal was 12 (range 4–56) days for morphine and 48 (range 7–57) days for placebo]. All continued to provide outcome data. Participants were asked to take two capsules a day for 84 days, which totalled to 168 tablets. Estimates of the proportion of tablets taken ranged from 39–51% in those assigned to morphine and 64–83% in those assigned to placebo, depending on an assumption that none or all the pills were taken if bottles were not returned.

There were 12 serious adverse events in the morphine group and 15 in the placebo group (Supporting Information, *Table* *S4A*). One death occurred in the placebo group. One morphine group participant had a marked cognitive decline from baseline at week 4 (25 to 14 MoCA points) that coincided with a decline in renal function. The patient fully recovered after stopping morphine. Non‐serious adverse events (Supporting Information, *Table* *S4B*) were more common in those assigned to morphine (32 events) compared with placebo (22 events), although the excess was mainly due to one individual assigned to morphine who had nine non‐serious events

After randomization, up to and including week 4, 18 (86%) participants assigned to morphine and 13 (54%) to placebo reported at least one harm of grade 1 or more, and 10 (48%) and 1 (4.2%), respectively, of grade 2 or more (Supporting Information, *Table* *S5A*—up to week 4; Supporting Information, *Table* *S5B*—weeks 8 and 12). Constipation, nausea, and vomiting were more common in those assigned to morphine rather than placebo throughout the trial but were mainly mild (grade 1). Study laxative/placebo was not taken by a substantial number of participants. Treatment‐emergent adverse events during the first week were three times more common in the morphine group and were more common in participants with eGFR <54 mL/min (the mean value) (*Table* 5), but all apart from one were grade 2 or less. Most presented by day 4 (see Supporting Information, *Figure* *S1*). Harms by grade, treatment group, and time point to week 4 are presented in Supporting Information, *Tables S5A* and *S5B*.

**Table 5:** Number of participants experiencing a treatment‐emergent harm within the first week of follow‐up, stratified by median baseline eGFR of 54 mL/min

	Grade ≤ 2				Grade ≥ 3			
	Morphine		Placebo		Morphine		Placebo	
	eGFR ≤ 54 (*n* = 14)	eGFR > 54 (*n* = 7)	eGFR ≤ 54 (*n* = 9)	eGFR > 54 (*n* = 15)	eGFR ≤ 54 (*n* = 14)	eGFR > 54 (*n* = 7)	eGFR ≤ 54 (*n* = 9)	eGFR > 54 (*n* = 15)
Confusion	0 (0.0)	1 (14.3)	0 (0.0)	1 (6.7)	0 (0.0)	0 (0.0)	0 (0.0)	0 (0.0)
Constipation	9 (64.3)	5 (71.4)	0 (0.0)	2 (13.3)	0 (0.0)	0 (0.0)	0 (0.0)	0 (0.0)
Vomiting	5 (35.7)	1 (14.3)	1 (11.1)	0 (0.0)	0 (0.0)	0 (0.0)	0 (0.0)	0 (0.0)
Nausea	9 (64.3)	3 (42.9)	1 (11.1)	2 (13.3)	0 (0.0)	0 (0.0)	0 (0.0)	0 (0.0)
Memory impairment	1 (7.1)	1 (14.3)	0 (0.0)	1 (6.7)	0 (0.0)	0 (0.0)	0 (0.0)	0 (0.0)
Cognitive disturbance	0 (0.0)	0 (0.0)	0 (0.0)	0 (0.0)	1 (7.1)	0 (0.0)	0 (0.0)	0 (0.0)
At least one	11 (78.6)	6 (85.7)	1 (11.1)	5 (33.3)	1 (7.1)	0 (0.0)	0 (0.0)	0 (0.0)

Health service use and EQ‐5D‐5L measures at baseline and follow‐up are presented in Supporting Information, *Tables S6A and S6B*.

## Discussion

### Main findings/results of the study

The BreatheMOR‐HF trial is the first to provide placebo‐controlled data for medium‐duration, modified release, steady state, low dose, oral morphine for people with persistent breathlessness despite guideline‐recommended treatments for chronic heart failure. The trial failed to enrol the planned number of participants but provides valuable insights into the potential rate and severity of morphine‐related harm, particularly pertinent given the recent license extension to chronic breathlessness (including that due to heart failure) for a sustained release oral morphine preparation (Kapanol™) by the Therapeutic Goods Administration in Australia. Constipation, nausea, and vomiting, albeit mainly mild, were more common in the morphine group, as was study drug withdrawal. This highlights the need for early skilful management of morphine‐related side effects and careful clinical decision making regarding prescription of morphine for chronic breathlessness given the persisting lack of robust evidence of benefit in this patient population.

### Strengths and limitations

The major limitation of the study is its early termination and consequent lack of power; data can only be interpreted as preliminary. Recruitment challenges are related to (i) some eligibility criteria, particularly the natriuretic peptide threshold, (ii) the Research Ethics Committee requirement that participants avoided driving for the first week (despite no evidence base^37^), and (iii) delays in opening recruitment sites. Suboptimal adherence to, and withdrawal from, study drug weakened our findings. Numbers are too small for a per protocol analysis, but inclusion of data from those who stopped study drug may have diluted benefit experienced by those who tolerated morphine.

The major strength is the double‐blind, placebo‐controlled design and trial duration. Data quality and completion rates (apart from physical activity and exercise tolerance) were very high, with minimal full study withdrawal. In addition, our study recruited the targeted population with advanced disease.

### What this study adds

Although participants had advanced disease, there were no excess serious adverse events in the morphine group. We found no respiratory depression consistent with a recent systematic review and meta‐analysis.^17^, ^18^

The reports of (mainly mild) constipation, nausea, and vomiting in the morphine group are similar to other published reports of low dose morphine for breathlessness^14^, ^15^, ^38^, ^39^ but are nonetheless important. In this study protocol, anti‐emetics were not co‐prescribed from study drug initiation in the same way as laxatives but given in response to emergent nausea. It is possible that patients with heart failure and renal dysfunction may be susceptible to nausea as blood brain barrier permeability is increased, at least in acute kidney injury,^40^ and initial co‐prescription might be useful. Recommendations for management of morphine‐related side effects are available but may be unfamiliar to non‐palliative care or non‐pain specialists.^41^

Impaired cognition is cited as a particular fear of morphine treatment by both patients and clinicians,^42^ but we found no excess sleepiness or cognitive impairment in the morphine group apart from one patient with deterioration in renal function. We saw a reduction in daily steps with morphine but no increase in daytime sleepiness. The walk distance increased further in the morphine group, but there was a high proportion of missing data making interpretation difficult. The lack of desaturation on exertion is reassuring and consistent with previous findings.^17^

In the morphine group, all breathlessness measures, apart from week 4 average breathlessness, had greater improvement from baseline than the placebo group. Improvements in all breathlessness measures were sustained or improved further by weeks 8 and 12 in the morphine group and reached clinically important differences. Further improvement beyond week 4 was not seen in the placebo group in any breathlessness measure, and none reached clinical significance. At baseline, the breathlessness scores were on average worse in the morphine group than control by around a clinically important difference (1 point) for each measure and so may represent a group more likely to respond to morphine.^43^ However, such findings can only be interpreted as a preliminary signal of benefit.

### Implications for clinical practice and research

Morphine should only be prescribed in people with heart failure when other measures have not helped and only with early recourse to management of potential side effects. Fears of serious harm are unsubstantiated.

The observed pattern of improvement in breathlessness measures in the morphine group suggests that an adequately sized trial would be useful. Lessons learned from recruitment and attrition challenges should be incorporated in a new study. A dose titration step should be included, and an initiation side‐effect management plan should be put in place. The eligibility criterion relating to natriuretic peptide should be removed but included as a secondary outcome in view of the observation that levels reduced by more in the morphine group, a finding seen in previous work and the significance of which is unknown.^14^ The extensive trials unit support required to navigate the complex governance required to open multiple recruitment sites needs to be planned for.

The observed standard deviation of the primary outcome measure was lower than the anticipated 2.55 in each group at all time points; the correlation between the baseline and week 4 measures of the primary endpoint was 0.67. A recalculated sample size of 150 patients would provide 80% power to detect the same planned difference, assuming a standard deviation of 2.55, 5% significance level, a conservative correlation of 0.65 between the baseline and week 4 measures, and 20% attrition.

## Conclusions

We were unable to answer our primary objectives due to inadequate power. However, we provide novel preliminary placebo‐controlled data relating to the benefit and safety of medium‐term oral modified release morphine that will help inform clinical practice and the design of a future trial.

## Conflict of interest

D.C.C. has received an unrestricted research grant from Mundipharma, is an unpaid member of an advisory board for Helsinn Pharmaceuticals, and has consulted Mayne Pharma and received intellectual property payments from them. M.J.J. has received consulting payments from Mayne Pharma. No authors have any conflicting interests with the content of this manuscript.

## Author contributions

M.J., D.C.C., and A.L.C. did the concept and design. Protocol authors were M.J.J., D.C.C., J.C., A.L.C., R.G., K.H., J.M., S.O., G.R., D.T., V.A., and S.C. Trial conduct and data management were performed by S.C., K.B., L.J., and K.H. Data collection was carried out by K.H., S.O., J.G., M.J.J., and A.L.C. C.F., R.G., and V.A. analysed the data. All authors did the data interpretation, revised the manuscript for intellectual content, and approved the final version.

## Funding

This work was supported by the British Heart Foundation (grant number CS/13/2/30584).

## Data management and sharing

Requests to access BreatheMOR‐HF data can be made to the corresponding author. Requests will be considered on a case‐by‐case basis and managed according to the York Trials Unit, University of York processes and procedures. The full protocol can be accessed through the corresponding author.

## Supporting information


**Table S1**. Global Impression of Change relating to breathlessness, at week 4 (valid data provided by 41 participants).
**Table S2**. Clinical assessments and NYHA class at week 4 by randomised group.
**Table S3**. Study drug use by randomised group
**Table S4**. (A) Serious adverse events. (B) Non‐serious adverse events.
**Table S5**. (A) Harms by grade, treatment group and time point, up to week 4. (B) Harms by grade, treatment group and time point, weeks 8 and 12.
**Table S6**. (A) EQ‐5D‐5L, and health service use during previous 4 weeks, at baseline and week 4 by randomised group. (B) EQ‐5D‐5L, and health service use during previous 4 weeks, at weeks 8 and 12 by randomised group.
**Figure S1**. Supporting information

## References

[1] Nordgren L , Sorensen S . Symptoms experienced in the last six months of life in patients with end‐stage heart failure. Eur J Cardiovasc Nurs 2003; 2: 213–217. 14622629 10.1016/S1474-5151(03)00059-8

[2] Currow DC , Dal GE , Ferreira D , Johnson MJ , McCaffrey N , Ekstrom M . Chronic breathlessness associated with poorer physical and mental health‐related quality of life (SF‐12) across all adult age groups. Thorax 2017; 72: 1151–1153. 28356419 10.1136/thoraxjnl-2016-209908

[3] Smith AK , Currow DC , Abernethy AP , Johnson MJ , Miao Y , Boscardin WJ , Ritchie CS . Prevalence and outcomes of breathlessness in older adults: a national population study. J Am Geriatr Soc 2016; 64: 2035–2041. 27603500 10.1111/jgs.14313

[4] Hutchinson A , Pickering A , Williams P , Bland JM , Johnson MJ . Breathlessness and presentation to the emergency department: a survey and clinical record review. BMC Pulm Med 2017; 17: 53. 28320369 10.1186/s12890-017-0396-4 PMC5360046

[5] Parshall MB . Adult emergency visits for chronic cardiorespiratory disease: does dyspnea matter? Nurs Res 1999; 48: 62–70. 10190832 10.1097/00006199-199903000-00004

[6] Farquhar MC , Prevost A , McCrone P , Brafman‐Price B , Bentley A , Higginson IJ , Todd C , Booth S . Is a specialist breathlessness service more effective and cost‐effective for patients with advanced cancer and their carers than standard care? Findings of a mixed‐method randomised controlled trial. BMC Med 2014; 12: 194. 25358424 10.1186/s12916-014-0194-2 PMC4222435

[7] Ekstrom M , Bajwah S , Bland JM , Currow DC , Hussain JA , Johnson MJ . One evidence base; three stories: do opioids relieve chronic breathlessness? Thorax 2018; 73: 88–90. 28377491 10.1136/thoraxjnl-2016-209868

[8] Higginson IJ , Bausewein C , Reilly CC , Gao W , Gysels M , Dzingina M , McCrone P , Booth S , Jolley CJ , Moxham J . An integrated palliative and respiratory care service for patients with advanced disease and refractory breathlessness: a randomised controlled trial. Lancet Respir Med 2014; 2: 979–987. 25465642 10.1016/S2213-2600(14)70226-7

[9] von Leupoldt A , Sommer T , Kegat S , Baumann HJ , Klose H , Dahme B , Büchel C . Dyspnea and pain share emotion‐related brain network. Neuroimage 2009; 48: 200–206. 19527787 10.1016/j.neuroimage.2009.06.015

[10] Baumgartner U , Buchholz HG , Bellosevich A , Magerl W , Siessmeier T , Rolke R , Höhnemann S , Piel M , Rösch F , Wester HJ , Henriksen G . High opiate receptor binding potential in the human lateral pain system. Neuroimage 2006; 30: 692–699. 16337817 10.1016/j.neuroimage.2005.10.033

[11] Gifford AH , Mahler DA , Waterman LA , Ward J , Kraemer WJ , Kupchak BR , Baird JC . Neuromodulatory effect of endogenous opioids on the intensity and unpleasantness of breathlessness during resistive load breathing in COPD. COPD 2011; 8: 160–166. 21513438 10.3109/15412555.2011.560132

[12] Mahler DA , Murray JA , Waterman LA , Ward J , Kraemer WJ , Zhang X , Baird JC . Endogenous opioids modify dyspnoea during treadmill exercise in patients with COPD. Eur Respir J 2009; 33: 771–777. 19213787 10.1183/09031936.00145208

[13] Barnes H , McDonald J , Smallwood N , Manser R . Opioids for the palliation of refractory breathlessness in adults with advanced disease and terminal illness. Cochrane Database Syst Rev 2016; 3: CD011008. 27030166 10.1002/14651858.CD011008.pub2 PMC6485401

[14] Johnson MJ , McDonagh TA , Harkness A , McKay SE , Dargie HJ . Morphine for the relief of breathlessness in patients with chronic heart failure‐a pilot study. Eur J Heart Fail 2002; 4: 753–756. 12453546 10.1016/s1388-9842(02)00158-7

[15] Oxberry SG , Torgerson DJ , Bland JM , Clark AL , Cleland JG , Johnson MJ . Short‐term opioids for breathlessness in stable chronic heart failure: a randomized controlled trial. Eur J Heart Fail 2011; 13: 1006–1012. 21712288 10.1093/eurjhf/hfr068

[16] Oxberry SG , Bland JM , Clark AL , Cleland JG , Johnson MJ . Repeat dose opioids may be effective for breathlessness in chronic heart failure if given for long enough. J Palliat Med 2013; 16: 250–255. 23368980 10.1089/jpm.2012.0270

[17] Verberkt CA , van den Beuken‐van Everdingen MHJ , Schols JMGA , Datla S , Dirksen CD , Johnson MJ , van Kuijk SM , Wouters EF , Janssen DJ . Respiratory adverse effects of opioids for breathlessness: a systematic review and meta‐analysis. Eur Respir J 2017; 50: 1701153. 29167300 10.1183/13993003.01153-2017

[18] Rocker GM , Young J , Horton R. Using opioids to treat dyspnea in advanced COPD: a survey of Canadian clinicians. Chest (accessible at http://chestnet.org). 2008. Ref Type: Abstract

[19] Cockcroft DW , Gault MH . Prediction of creatinine clearance from serum creatinine. Nephron 1976; 16: 31–41. 1244564 10.1159/000180580

[20] Charlson M , Szatrowski TP , Peterson J , Gold J . Validation of a combined comorbidity index. J Clin Epidemiol 1994; 47: 1245–1251. 7722560 10.1016/0895-4356(94)90129-5

[21] Stenton C . The MRC breathlessness scale. Occup Med (Lond) 2008; 58: 226–227. 18441368 10.1093/occmed/kqm162

[22] Gift AG , Narsavage G . Validity of the numeric rating scale as a measure of dyspnea. Am J Crit Care 1998; 7: 200–204. 9579246

[23] Jaeschke R , Singer J , Guyatt GH . Measurement of health status. Ascertaining the minimal clinically important difference. Control Clin Trials 1989; 10: 407–415. 2691207 10.1016/0197-2456(89)90005-6

[24] Breivik H , Borchgrevink PC , Allen SM , Rosseland LA , Romundstad L , Hals EK , Kvarstein G , Stubhaug A . Assessment of pain. Br J Anaesth 2008; 101: 17–24. 18487245 10.1093/bja/aen103

[25] Johns MW . A new method for measuring daytime sleepiness: the Epworth sleepiness scale. Sleep 1991; 14: 540–545. 1798888 10.1093/sleep/14.6.540

[26] Ingle L , Shelton RJ , Rigby AS , Nabb S , Clark AL , Cleland JG . The reproducibility and sensitivity of the 6‐min walk test in elderly patients with chronic heart failure. Eur Heart J 2005; 26: 1742–1751. 15831556 10.1093/eurheartj/ehi259

[27] Grant PM , Ryan CG , Tigbe WW , Granat MH . The validation of a novel activity monitor in the measurement of posture and motion during everyday activities. Br J Sports Med 2006; 40: 992–997. 16980531 10.1136/bjsm.2006.030262 PMC2577473

[28] Gallagher R , Sullivan A , Burke R , Hales S , Gillies G , Cameron J , Saliba B , Tofler G . Mild cognitive impairment, screening, and patient perceptions in heart failure patients. J Card Fail 2013; 19: 641–646. 24054341 10.1016/j.cardfail.2013.08.001

[29] The Criteria Committee of the New York Heart Association . Nomenclature and criteria for diagnosis of diseases of the heart and great vessels. 6th ed. Boston, Mass: Little, Brown & Co; 1994.

[30] Abernethy AP , Shelby‐James T , Fazekas BS , Woods D , Currow DC . The Australia‐modified Karnofsky Performance Status (AKPS) scale: a revised scale for contemporary palliative care clinical practice [ISRCTN81117481]. BMC Palliat Care 2005; 4: 7. 16283937 10.1186/1472-684X-4-7 PMC1308820

[31] Jones P , Gosch K , Yi L , Reid K , Tang F , Chan P , Spertus J . The KCCQ‐12: A short version of the Kansas City Cardiomyopathy Questionnaire. Circ Cardiovasc Qual Outcomes 2013; 6: A248. 10.1161/CIRCOUTCOMES.115.001958 PMC4885562 26307129

[32] National Institute for Health, National Cancer Institute . Common terminology criteria for adverse events version 4.03. 1‐6‐2010. US Department of Health and Human Services. 21‐7‐2018. Ref Type: Report

[33] Kaida K , Takahashi M , Akerstedt T , Nakata A , Otsuka Y , Haratani T , Fukasawa K . Validation of the Karolinska sleepiness scale against performance and EEG variables. Clin Neurophysiol 2006; 117: 1574–1581. 16679057 10.1016/j.clinph.2006.03.011

[34] Kind P . The EuroQoL instrument: an index of health‐related quality of life In Spilker B., ed. Quality of life and pharmacoeconomics in clinical trials. Philadelphia: Lippincott‐Raven; 1996.

[35] Akaike H . A new look at the statistical model identification. IEEE Trans Autom Control 1974; 19: 716–723.

[36] Rennie D . CONSORT revised—improving the reporting of randomized trials. JAMA 2001; 285: 2006–2007. 11308440 10.1001/jama.285.15.2006

[37] Ferreira DH , Boland JW , Phillips JL , Lam L , Currow DC . The impact of therapeutic opioid agonists on driving‐related psychomotor skills assessed by a driving simulator or an on‐road driving task: a systematic review. Palliat Med 2018; 32: 786–803. 29299954 10.1177/0269216317746583

[38] Abernethy AP , Currow DC , Frith P , Fazekas BS , McHugh A , Bui C . Randomised, double blind, placebo controlled crossover trial of sustained release morphine for the management of refractory dyspnoea. BMJ 2003; 327: 523–528. 12958109 10.1136/bmj.327.7414.523 PMC192892

[39] Currow DC , McDonald C , Oaten S , Kenny B , Allcroft P , Frith P , Briffa M , Johnson MJ , Abernethy AP . Once‐daily opioids for chronic dyspnea: a dose increment and pharmacovigilance study. J Pain Symptom Manage 2011; 42: 388–399. 21458217 10.1016/j.jpainsymman.2010.11.021

[40] Nongnuch A , Panorchan K , Davenport A . Brain‐kidney crosstalk. Crit Care 2014; 18: 225. 25043644 10.1186/cc13907 PMC4075125

[41] Cherny N , Ripamonti C , Pereira J , Davis C , Fallon M , McQuay H , Mercadante S , Pasternak G , Ventafridda V , for the Expert Working Group of the European Association of Palliative Care Network . Strategies to manage the adverse effects of oral morphine: an evidence‐based report. J Clin Oncol 2001; 19: 2542–2554. 11331334 10.1200/JCO.2001.19.9.2542

[42] Jacobsen R , Liubarskiene Z , Moldrup C , Christrup L , Sjogren P , Samsanaviciene J . Barriers to cancer pain management: a review of empirical research. Medicina (Kaunas) 2009; 45: 427–433. 19605961

[43] Johnson MJ , Bland JM , Oxberry SG , Abernethy AP , Currow DC . Opioids for chronic refractory breathlessness: patient predictors of beneficial response. Eur Respir J 2013; 42: 758–766. 23258776 10.1183/09031936.00139812

