# Supplementary material for: Oral modified release morphine for breathlessness in chronic heart failure: a randomized placebo‐controlled trial
Source: ESC Heart Fail. 2019 Aug 6;6(6):1149–60. doi: 10.1002/ehf2.12498 (PMC6989293; doi:10.1002/ehf2.12498)
Supplement: Supplementary file 1 — Table S1. Global Impression of Change relating to breathlessness, at week 4 (valid data provided by 41 participants). Table S2. Clinical assessments and NYHA class at week 4 by randomised group. Table S3. Study drug use by randomised group Table S4. (A) Serious adverse events. (B) Non‐serious adverse events. Table S5. (A) Harms by grade, treatment group and time point, up to week 4. (B) Harms by grade, treatment group and time point, weeks 8 and 12. Table S6. (A) EQ‐5D‐5L, and health service use during previous 4 weeks, at baseline and week 4 by randomised group. (B) EQ‐5D‐5L, and health service use during previous 4 weeks, at weeks 8 and 12 by randomised group. Figure S1. Supporting information [file EHF2-6-1149-s001.docx]

Supplementary Table 1: Global Impression of Change relating to breathlessness, at week 4 (valid data provided by 41 participants)

| **Global Impression of Change** | **Morphine**  **(n=20)** | **Placebo**  **(n=21)** | **Total**  **(n=41)** |
| --- | --- | --- | --- |
| **Is your breathing** |  |  |  |
| Worse | 1 (5.0) | 1 (4.8) | 2 (4.9) |
| About the same | 13 (65.0) | 9 (42.9) | 22 (53.7) |
| Better | 6 (30.0) | 11 (52.4) | 17 (41.5) |
| If better… |  |  |  |
| **If your breathing is better, how much better is your breathing?** | **N=6** | **N=11** | **N=17** |
| Almost the same, hardly any better at all | 0 (0.0) | 0 (0.0) | 0 (0.0) |
| A little better | 3 (50.0) | 4 (36.4) | 7 (41.2) |
| Somewhat better | 1 (16.7) | 2 (18.2) | 3 (17.7) |
| Moderately better | 0 (0.0) | 2 (18.2) | 2 (11.7) |
| A good deal better | 2 (33.3) | 2 (18.2) | 4 (23.5) |
| A great deal better | 0 (0.0) | 1 (9.1) | 1 (5.9) |
| A very great deal better | 0 (0.0) | 0 (0.0) | 0 (0.0) |
| **If your breathing is worse, how much worse is your breathing?** | **N=1** | **N=1** | **N=2** |
| Almost the same, hardly any worse at all | 0 (0.0) | 0 (0.0) | 0 (0.0) |
| A little worse | 0 (0.0) | 0 (0.0) | 0 (0.0) |
| Somewhat worse | 0 (0.0) | 1 (100.0) | 1 (50.0) |
| Moderately worse | 0 (0.0) | 0 (0.0) | 0 (0.0) |
| A good deal worse | 1 (100.0) | 0 (0.0) | 1 (50.0) |
| A great deal worse | 0 (0.0) | 0 (0.0) | 0 (0.0) |
| A very great deal worse | 0 (0.0) | 0 (0.0) | 0 (0.0) |

**Supplementary Table 2: Clinical assessments and NYHA class at week 4 by randomised group**

| **Characteristic** | **Morphine**  **(n=21)** | **Placebo**  **(n=24)** | **Total**  **(n=45)** |
| --- | --- | --- | --- |
| NYHA Class  *II*  *III*  *IV*  *Missing* | 1 (4.8)  18 (85.7)  1 (4.8)  1 (4.8) | 1 (4.2)  21 (87.5)  0 (0.0)  2 (8.3) | 2 (4.4)  39 (86.7)  1 (2.2)  3 (6.7) |
| Resting pulse rate (per minute) (radial) | 69.5 (11.8) | 72.1 (9.6) | 70.9 (10.6) |
| Resting systolic blood pressure, mmHg | 109.4 (16.4) | 111.5 (19.3) | 110.5 (17.8) |
| Resting diastolic blood pressure, mmHg | 62.9 (9.0) | 65.4 (12.0) | 64.2 (10.6) |
| Resting respiratory rate (per minute) | 16.6 (6.0) | 15.3 (3.4) | 15.9 (4.8) |
| Pulse Oximetry, % | 96.3 (2.1) | 97.0 (2.0) | 96.7 (2.0) |
| NTproBNP^c^, pg/mL | 2169 (1092, 3851) | 2851 (1694, 5437) | 2598 (1092, 4982) |
| ^a^ Continuous data is presented as mean (SD) or median (IQR), and categorical data as n (%); ^b^ NTproBNP conducted by certain sites only | | | |

**Supplementary Table 3**

Data on study drug use is described here, including estimates of number of pills taken as determined by number unused and returned. The cases where pills were dispensed but the bottles not returned were managed in two ways, assuming: i) that all the pills in that batch were taken; and ii) that none were taken.

The first dose was taken a median of 1 day after randomisation in the morphine group (range 0 to 7), and 0.5 days in the placebo group (range 0 to 5). Most first doses were taken in the afternoon (morphine group, n=18, 90.0%; placebo group, n=21, 87.5%).

It was intended for participants to take two IMP capsules a day for 84 days, a total of 168 tablets. Estimates of the proportion of tablets taken range from 39% to 51% in the morphine group, and 64% to 83% in the placebo group, depending on whether it is assumed that no or all the pills were taken in cases where bottles were not returned (Supplementary Table **5**).

**Supplementary Table 3: Study drug use by randomised group**

|  | **Morphine/Placebo** | | **Docusate/Placebo** | |
| --- | --- | --- | --- | --- |
|  | **Morphine**  **(n=21)** | **Placebo**  **(n=24)** | **Morphine**  **(n=21)** | **Placebo**  **(n=24)** |
| **Dispensed at, n (%):**  Baseline  Week 4  Week 8 | 21 (100.0)  13 (61.9)  9 (42.9) | 24 (100.0)  22 (91.7)  17 (70.8) | 21 (100.0)  13 (61.9)  9 (42.9) | 24 (100.0)  22 (91.7)  17 (70.8) |
| **Total number dispensed**  Mean (SD)  Median (min, max) | 114.7 (51.6)  112 (56, 168) | 147.0 (36.2)  168 (56, 168) | 114.7 (51.6)  112 (56, 168) | 147.0 (36.2)  168 (56, 168) |
| **Assume all pills taken if return pill count missing** | | | | |
| **Total number returned**  Mean (SD)  Median (min, max) | 29.8 (24.6)  37 (0, 91) | 17.9 (22.1)  6 (0, 64) | 41.8 (39.5)  39 (0, 168) | 32.1 (41.1)  12.5 (0, 168) |
| **Percentage taken of drugs taken that were dispensed**  Mean (SD)  Median (min, max) | 59.6 (36.3)  67.0 (0, 100) | 82.6 (26.4)  96.4 (5.4, 100) | 52.4 (36.3)  44.0 (0, 100) | 73.2 (34.1)  90.9 (0, 100) |
| **Total used out of number intended (n=168)**  Mean (SD)  Median (min, max) | 50.5 (42.0)  44.6 (0, 100) | 76.9 (31.3)  96.1 (1.8, 100) | 43.4 (40.4)  33.3 (0, 100) | 68.4 (36.2)  86.6 (0, 100) |
| **Assume no pills taken if return pill count missing** | | | | |
| **Total number returned**  Mean (SD)  Median (min, max) | 48.4 (30.6)  46 (0, 118) | 38.9 (42.1)  32.5 (0, 168) | 55.1 (41.0)  46 (0, 168) | 53.1 (51.8)  43.5 (0, 168) |
| **Percentage taken of drugs taken that were dispensed**  Mean (SD)  Median (min, max) | 48.5 (30.1)  35.7 (0, 100) | 69.4 (32.8)  79.5 (0, 100) | 44.5 (31.4)  33.3 (0, 100) | 59.3 (38.0)  69.3 (0, 100) |
| **Total used out of number intended (n=168)**  Mean (SD)  Median (min, max) | 39.4 (33.8)  33.3 (0, 100) | 64.4 (34.8)  69.6 (0, 100) | 35.5 (33.9)  29.8 (0, 100) | 55.9 (39.1)  64.9 (0, 100) |

**Supplementary Tables 4a and 4b**

Supplementary Table 4a: Serious adverse events

| **Serious adverse events** | **Morphine**  **(n=21)** | **Placebo**  **(n=24)** | **Total**  **(n=45)** |
| --- | --- | --- | --- |
| **Number of events** | 12 | 15 | 27 |
| **Number of participants with ≥1 event, n (%)**^a^ | 7 (33.3) | 10 (41.7) | 17 (37.8) |
| **Number of events/participant, n (%)^b^**  1  2  3  4 | 3 (42.9)  3 (42.9)  1 (14.3)  0 (0.0) | 8 (80.0)  0 (0.0)  1 (10.0)  1 (10.0) | 11 (64.7)  3 (17.7)  2 (11.8)  1 (5.9) |
| **Type of event, n (%)^c^**  Death  Life-threatening  Disability/incapacity  Hospitalisation  Prolonged hospital stay  Congenital anomaly  Other^d^ | 0 (0.0)  0 (0.0)  0 (0.0)  10 (83.3)  0 (0.0)  0 (0.0)  2 (16.7) | 1 (6.7)  0 (0.0)  0 (0.0)  14 (93.3)  0 (0.0)  0 (0.0)  0 (0.0) | 1 (3.7)  0 (0.0)  0 (0.0)  24 (88.9)  0 (0.0)  0 (0.0)  2 (7.4) |
| **Severity, n (%)^c^**  Mild  Moderate  Severe  Missing | 0 (0.0)  7 (58.3)  2 (16.7)  3 (25.0) | 3 (20.0)  1 (6.7)  6 (40.0)  5 (33.3) | 3 (11.1)  8 (29.6)  8 (29.6)  8 (29.6) |
| **Relatedness to morphine, n (%)^c^**  Not related  Unlikely to be related  Possibly related  Probably related  Definitely related | 8 (66.7)  1 (8.3)  2 (16.7)  1 (8.3)  0 (0.0) | 12 (80.0)  2 (13.3)  0 (0.0)  1 (6.7)  0 (0.0) | 20 (74.1)  3 (11.1)  2 (7.4)  2 (7.4)  0 (0.0) |
| **Relatedness to docusate, n (%)^c^**  Not related  Unlikely to be related  Possibly related  Probably related  Definitely related | 10 (83.3)  2 (16.7)  0 (0.0)  0 (0.0)  0 (0.0) | 14 (93.3)  1 (6.7)  0 (0.0)  0 (0.0)  0 (0.0) | 24 (88.9)  3 (11.1)  0 (0.0)  0 (0.0)  0 (0.0) |
| **Expectedness, n (%)^e^**  Expected  Not expected^f^ | 2 (66.7)  1 (33.3) | 1 (100.0)  0 (0.0) | 3 (75.0)  1 (25.0) |
| ^a^ percentage out of number of randomised participants; ^b^ percentages out of number of participants with at least one event; ^c^ percentage out of number of events; ^d^ cognitive decline (n=1); slight deterioration in mental health state (n=1); ^e^ only if event possibly, probably or definitely related to morphine, percentage out of number of these events; ^f^ this event was reported to the MHRA as a SUSAR and the participants allocation was unblinded to the treating clinician (marked cognitive decline noted whilst performing MoCA outcome at week 4 time point). | | | |

A total of 54 non-serious adverse events were reported for 26 participants (14 (67%) in the morphine group, and 12 (50%) in the placebo group). Half of these participants experienced more than one event (range 1 to 9). Nineteen events were deemed to be at least possibly related to the IMP (16 (50%) in the morphine group, and 3 (14%) in the placebo group), of which only one was unexpected (participant reported dry month of mild intensity). The most commonly reported events were nausea (6 occurrences for 5 morphine participants, and 1 occurrence for 1 placebo participant), cognitive disturbance (5 occurrences for 3 morphine participants), and lung infection (1 occurrence for 1 morphine participant, and 4 occurrences for 4 placebo participants).

Supplementary Table 4b: Non-serious adverse events

| **Non-serious adverse events** | **Morphine**  **(n=21)** | **Placebo**  **(n=24)** | **Total**  **(n=45)** |
| --- | --- | --- | --- |
| **Number of events** | 32 | 22 | 54 |
| **Number of participants with ≥1 event, n (%)**^a^ | 14 (66.7) | 12 (50.0) | 26 (57.8) |
| **Number of events/participant, n (%)^b^**  1  2  3  4  5  …  9 | 7 (50.0)  3 (21.4)  2 (14.3)  1 (7.1)  0 (0.0)  0 (0.0)  1 (7.1) | 6 (50.0)  4 (33.3)  1 (8.3)  0 (0.0)  1 (8.3)  0 (0.0)  0 (0.0) | 13 (50.0)  7 (26.9)  3 (11.5)  1 (3.9)  1 (3.9)  0 (0.0)  1 (3.9) |
| **Severity, n (%)^c^**  Mild  Moderate  Severe  Missing | 14 (43.8)  17 (53.1)  1 (3.1)  0 (0.0) | 11 (50.0)  8 (36.4)  1 (4.6)  2 (9.1) | 25 (46.3)  25 (46.3)  2 (3.7)  2 (3.7) |
| **Relatedness to IMP, n (%)^c^**  Not related  Unlikely to be related  Possibly related  Probably related  Definitely related | 11 (34.3)  5 (15.6)  9 (28.1)  5 (15.6)  2 (6.3) | 12 (54.6)  7 (31.8)  3 (13.6)  0 (0.0)  0 (0.0) | 23 (42.6)  12 (22.2)  12 (22.2)  5 (9.3)  2 (3.7) |
| **Relatedness to NIMP, n (%)^c^**  Not related  Unlikely to be related  Possibly related  Probably related  Definitely related | 23 (71.9)  5 (15.6)  4 (12.5)  0 (0.0)  0 (0.0) | 15 (68.2)  5 (22.7)  2 (9.1)  0 (0.0)  0 (0.0) | 38 (70.4)  10 (18.5)  6 (11.1)  0 (0.0)  0 (0.0) |
| **Expectedness for IMP, n (%)**  Expected  Not expected  Missing | 19 (59.4)  12 (37.5)  1 (3.1) | 5 (22.7)  15 (68.2)  2 (9.1) | 24 (44.4)  27 (50.0)  3 (5.6) |
| **Expectedness for NIMP, n (%)**  Expected  Not expected  Missing | 8 (25.0)  18 (56.2)  6 (18.8) | 3 (13.6)  17 (77.3)  2 (9.1) | 11 (20.4)  35 (64.8)  8 (14.8) |
| ^a^ percentage out of number of randomised participants; ^b^ percentages out of number of participants with at least one event; ^c^ percentage out of number of events | | | |

Supplementary Table 5a: Harms by grade, treatment group and time point, up to week 4

| **Harm symptom grade** | **Baseline** | | **Day 2** | | **Day 4** | | **Day 7** | | **Week 2** | | **Week 3** | | **Week 4** | |
| --- | --- | --- | --- | --- | --- | --- | --- | --- | --- | --- | --- | --- | --- | --- |
|  | **Morphine** | **Placebo** | **Morphine** | **Placebo** | **Morphine** | **Placebo** | **Morphine** | **Placebo** | **Morphine** | **Placebo** | **Morphine** | **Placebo** | **Morphine** | **Placebo** |
| **Confusion** |  |  |  |  |  |  |  |  |  |  |  |  |  |  |
| 0 | 20 (95.2) | 22 (91.7) | 19 (95.0) | 23 (95.8) | 19 (95.0) | 24 (100) | 19 (95.0) | 22 (91.7) | 18 (90.0) | 22 (91.7) | 20 (100) | 21 (91.3) | 18 (90.0) | 21 (91.3) |
| 1 | 1 (4.8) | 2 (8.3) | 1 (5.0) | 1 (4.2) | 0 (0.0) | 0 (0.0) | 0 (0.0) | 2 (8.3) | 2 (10.0) | 2 (8.3) | 0 (0.0) | 2 (8.7) | 2 (10.0) | 2 (8.7) |
| 2+ | 0 (0.0) | 0 (0.0) | 0 (0.0) | 0 (0.0) | 1 (5.0) | 0 (0.0) | 1 (5.0) | 0 (0.0) | 0 (0.0) | 0 (0.0) | 0 (0.0) | 0 (0.0) | 0 (0.0) | 0 (0.0) |
| **Constipation** |  |  |  |  |  |  |  |  |  |  |  |  |  |  |
| 0 | 18 (85.7) | 19 (79.2) | 13 (65.0) | 22 (91.7) | 10 (50.0) | 22 (91.7) | 7 (35.0) | 22 (91.7) | 14 (70.0) | 22 (91.7) | 15 (75.0) | 22 (95.7) | 14 (70.0) | 21 (91.3) |
| 1 | 3 (14.3) | 5 (20.8) | 7 (35.0) | 2 (8.3) | 7 (35.0) | 2 (8.3) | 9 (45.0) | 2 (8.3) | 6 (30.0) | 2 (8.3) | 4 (20.0) | 1 (4.3) | 6 (30.0) | 2 (8.7) |
| 2+ | 0 (0.0) | 0 (0.0) | 0 (0.0) | 0 (0.0) | 3 (15.0) | 0 (0.0) | 4 (20.0) | 0 (0.0) | 0 (0.0) | 0 (0.0) | 1 (5.0) | 0 (0.0) | 0 (0.0) | 0 (0.0) |
| **Vomiting** |  |  |  |  |  |  |  |  |  |  |  |  |  |  |
| 0 | 21 (100) | 24 (100) | 18 (90.0) | 24 (100) | 17 (85.0) | 24 (100) | 16 (80.0) | 23 (95.8) | 19 (95.0) | 24 (100) | 18 (90.0) | 23 (100) | 17 (85.0) | 22 (95.7) |
| 1 | 0 (0.0) | 0 (0.0) | 2 (10.0) | 0 (0.0) | 3 (15.0) | 0 (0.0) | 3 (15.0) | 1 (4.2) | 1 (5.0) | 0 (0.0) | 2 (10.0) | 0 (0.0) | 2 (10.0) | 1 (4.3) |
| 2+ | 0 (0.0) | 0 (0.0) | 0 (0.0) | 0 (0.0) | 0 (0.0) | 0 (0.0) | 1 (5.0) | 0 (0.0) | 0 (0.0) | 0 (0.0) | 0 (0.0) | 0 (0.0) | 1 (5.0) | 0 (0.0) |
| **Nausea** |  |  |  |  |  |  |  |  |  |  |  |  |  |  |
| 0 | 20 (95.2) | 23 (95.8) | 14 (70.0) | 23 (95.8) | 12 (60.0) | 21 (87.5) | 14 (70.0) | 21 (87.5) | 13 (65.0) | 23 (95.8) | 16 (80.0) | 22 (95.7) | 16 (80.0) | 18 (78.3) |
| 1 | 1 (4.8) | 1 (4.2) | 5 (25.0) | 1 (4.2) | 7 (35.0) | 2 (8.3) | 4 (20.0) | 3 (12.5) | 7 (35.0) | 1 (4.2) | 3 (15.0) | 1 (4.3) | 3 (15.0) | 5 (21.7) |
| 2+ | 0 (0.0) | 0 (0.0) | 1 (5.0) | 0 (0.0) | 1 (5.0) | 1 (4.2) | 2 (10.0) | 0 (0.0) | 0 (0.0) | 0 (0.0) | 1 (5.0) | 0 (0.0) | 1 (5.0) | 0 (0.0) |
| **Memory impairment** |  |  |  |  |  |  |  |  |  |  |  |  |  |  |
| 0 | 19 (90.5) | 18 (75.0) | 18 (94.7) | 22 (91.7) | 19 (95.0) | 23 (95.8) | 20 (100) | 21 (87.5) | 19 (95.0) | 22 (91.7) | 19 (95.0) | 21 (91.3) | 17 (85.0) | 20 (87.0) |
| 1 | 2 (9.5) | 6 (25.0) | 1 (5.3) | 2 (8.3) | 1 (5.0) | 1 (4.2) | 0 (0.0) | 3 (12.5) | 1 (5.0) | 2 (8.3) | 1 (5.0) | 2 (8.7) | 3 (15.0) | 3 (13.0) |
| 2+ | 0 (0.0) | 0 (0.0) | 0 (0.0) | 0 (0.0) | 0 (0.0) | 0 (0.0) | 0 (0.0) | 0 (0.0) | 0 (0.0) | 0 (0.0) | 0 (0.0) | 0 (0.0) | 0 (0.0) | 0 (0.0) |
| **Cognitive disturbance** |  |  |  |  |  |  |  |  |  |  |  |  |  |  |
| 0 | 19 (90.5) | 23 (95.8) | 20 (100) | 23 (95.8) | 20 (100) | 24 (100) | 19 (95.0) | 24 (100) | 18 (90.0) | 23 (95.8) | 19 (100) | 23 (100) | 18 (90.0) | 22 (95.7) |
| 1 | 2 (9.5) | 1 (4.2) | 0 (0.0) | 1 (4.2) | 0 (0.0) | 0 (0.0) | 0 (0.0) | 0 (0.0) | 1 (5.0) | 1 (4.2) | 0 (0.0) | 0 (0.0) | 2 (10.0) | 1 (4.3) |
| 2+ | 0 (0.0) | 0 (0.0) | 0 (0.0) | 0 (0.0) | 0 (0.0) | 0 (0.0) | 1 (5.0) | 0 (0.0) | 1 (5.0) | 0 (0.0) | 0 (0.0) | 0 (0.0) | 0 (0.0) | 0 (0.0) |

Supplementary Table 5b: Harms by grade, treatment group and time point, weeks 8 and 12

| **Harm symptom grade** | **Week 8** | | **Week 12** | |
| --- | --- | --- | --- | --- |
|  | **Morphine** | **Placebo** | **Morphine** | **Placebo** |
| **Confusion** |  |  |  |  |
| 0 | 16 (80.0) | 21 (91.3) | 17 (85.0) | 19 (86.4) |
| 1 | 3 (15.0) | 1 (4.3) | 1 (5.0) | 3 (13.6) |
| 2+ | 1 (5.0) | 1 (4.3) | 2 (10.0) | 0 (0.0) |
| **Constipation** |  |  |  |  |
| 0 | 15 (75.0) | 22 (95.7) | 15 (75.0) | 17 (77.3) |
| 1 | 5 (25.0) | 1 (4.3) | 4 (20.0) | 5 (22.7) |
| 2+ | 0 (0.0) | 0 (0.0) | 1 (5.0) | 0 (0.0) |
| **Vomiting** |  |  |  |  |
| 0 | 18 (90.0) | 23 (100.0) | 17 (85.0) | 21 (95.5) |
| 1 | 2 (10.0) | 0 (0.0) | 2 (10.0) | 1 (4.5) |
| 2+ | 0 (0.0) | 0 (0.0) | 1 (5.0) | 0 (0.0) |
| **Nausea** |  |  |  |  |
| 0 | 16 (80.0) | 20 (87.0) | 12 (60.0) | 20 (90.9) |
| 1 | 3 (15.0) | 1 (4.3) | 5 (25.0) | 1 (4.5) |
| 2+ | 1 (5.0) | 2 (8.7) | 3 (15.0) | 1 (4.5) |
| **Memory impairment** |  |  |  |  |
| 0 | 17 (85.0) | 19 (82.6) | 16 (80.0) | 18 (85.7) |
| 1 | 2 (10.0) | 2 (8.7) | 2 (10.0) | 3 (14.3) |
| 2+ | 1 (5.0) | 2 (8.7) | 2 (10.0) | 0 (0.0) |
| **Cognitive disturbance** |  |  |  |  |
| 0 | 19 (95.0) | 22 (95.7) | 18 (90.0) | 19 (86.4) |
| 1 | 0 (0.0) | 0 (0.0) | 0 (0.0) | 3 (13.6) |
| 2+ | 1 (5.0) | 1 (4.3) | 2 (10.0) | 0 (0.0) |

**Supplementary Tables 6a and 6b**

Supplementary Table 6a: EQ-5D-5L, and health service use during previous 4 weeks, at baseline and week 4 by randomised group

| **EQ-5D-5L, and health service use during previous 4 weeks** | **Morphine** | **Placebo** | **Total** |
| --- | --- | --- | --- |
| ***Baseline*** | **N=21** | **N=24** | **N=45** |
| EQ-5D-5L index value, Mean (SD)  Median (min, max) | 0.59 (0.15)  0.63 (0.23, 0.77) | 0.61 (0.20)  0.65 (0.04, 0.81) | 0.60 (0.17)  0.64 (0.04, 0.81) |
| EQ-5D-5L VAS, Mean (SD)  Median (min, max) | 51.7 (18.1)  50 (5, 85) | 55.1 (13.7)  54 (30, 90) | 53.5 (15.8)  50 (5, 90) |
| *Overnight stays in hospital, n (%)* | 0 (0.0) | 2 (8.3) | 2 (4.4) |
| Total number of nights, Mean (SD)  Median (min, max) | - | 5.0 (5.7)  5 (1, 9) | 5.0 (5.7)  5 (1, 9) |
| *Outpatient appointment, n (%)* | 12 (57.1) | 14 (58.3) | 26 (57.8) |
| Total number of visits, Mean (SD)  Median (min, max) | 1.6 (0.9)  1 (1, 3) | 1.6 (1.2)  1 (1, 5) | 1.6 (1.0)  1 (1, 5) |
| *Contact with GP, n (%)* | 12 (57.1) | 7 (29.2) | 19 (42.2) |
| Total number of contacts, Mean (SD)  Median (min, max) | 1.1 (0.3)  1 (1, 2) | 1.1 (0.4)  1 (1, 2) | 1.1 (0.3)  1 (1, 2) |
| Number at surgery, Mean (SD)  Median (min, max) | 1.0 (0.4)  1 (0, 2) | 1.1 (0.4)  1 (1, 2) | 1.1 (0.4)  1 (0, 2) |
| Number at home, Mean (SD)  Median (min, max) | 0.1 (0.3)  0 (0, 1) | 0.0 (0.0)  0 (0, 0) | 0.05 (0.2)  0 (0, 1) |
| Number via telephone, Mean (SD)  Median (min, max) | 0.0 (0.0)  0 (0, 0) | 0.0 (0.0)  0 (0, 0) | 0.0 (0.0)  0 (0, 0) |
| *Treatment at A&E department, n (%)* | 0 (0.0) | 3 (12.5) | 3 (6.7) |
| Total number of visits, Mean (SD)  Median (min, max) | - | 1.0 (0.0)  1 (1, 1) | 1.0 (0.0)  1 (1, 1) |
| *Contact with a nurse, n (%)* | 10 (47.6) | 11 (45.8) | 21 (46.7) |
| Total number of contacts, Mean (SD)  Median (min, max) | 1.7 (0.9)  1 (1, 3) | 2.1 (1.2)  2 (1, 4) | 1.9 (1.1)  1 (1, 4) |
| Number at surgery, Mean (SD)  Median (min, max) | 1.0 (1.1)  1 (0, 3) | 1.1 (1.1)  1 (0, 4) | 1.0 (1.1)  1 (0, 4) |
| Number at home, Mean (SD)  Median (min, max) | 0.3 (0.5)  0 (0, 1) | 0.9 (1.2)  1 (0, 4) | 0.6 (1.0)  0 (0, 4) |
| Number via telephone, Mean (SD)  Median (min, max) | 0.4 (1.0)  0 (0, 3) | 0.1 (0.3)  0 (0, 1) | 0.2 (0.7)  0 (0, 3) |
| ***Week 4*** | **N=20** | **N=24** | **N=45** |
| EQ-5D-5L index value, Mean (SD)  Medan (min, max) | 0.64 (0.17)  0.67 (0.16, 0.88) | 0.64 (0.22)  0.68 (-0.13, 0.91) | 0.64 (0.19)  0.68 (-0.13, 0.91) |
| EQ-5D-5L VAS, Mean (SD)  Median (min, max) | 50.2 (20.7)  50 (20, 90) | 58.0 (17.8)  60 (20, 85) | 54.3 (19.4)  55 (20, 90) |
| *Overnight stays in hospital, n (%)* | 3 (15.0) | 2 (8.3) | 5 (11.4) |
| Total number of nights, Mean (SD)  Median (min, max) | 4.7 (3.5)  5 (1, 8) | 5.5 (6.4)  6 (1, 10) | 5.0 (4.1)  5 (1, 10) |
| *Outpatient appointment, n (%)* | 6 (30.0) | 4 (16.7) | 10 (22.7) |
| Total number of visits, Mean (SD)  Median (min, max) | 1.7 (0.8)  2 (1, 3) | 2.0 (1.4)  2 (1, 4) | 1.8 (1.0)  2 (1, 4) |
| *Contact with GP, n (%)* | 13 (65.0) | 10 (41.7) | 23 (52.3) |
| Total number of contacts, Mean (SD)  Median (min, max) | 1.3 (0.6)  1 (1, 3) | 1.1 (0.3)  1 (1, 2) | 1.2 (0.5)  1 (1, 3) |
| Number at surgery, Mean (SD)  Median (min, max) | 0.9 (0.6)  1 (0, 2) | 1.0 (0.0)  1 (1, 1) | 1.0 (0.5)  1 (0, 2) |
| Number at home, Mean (SD)  Median (min, max) | 0.0 (0.0)  0 (0, 0) | 0.0 (0.0)  0 (0, 0) | 0.0 (0.0)  0 (0, 0) |
| Number via telephone, Mean (SD)  Median (min, max) | 0.4 (0.7)  0 (0, 2) | 0.1 (0.3)  0 (0, 1) | 0.3 (0.5)  0 (0, 2) |
| *Treatment at A&E department, n (%)* | 3 (15.0) | 3 (12.5) | 6 (13.6) |
| Total number of visits, Mean (SD)  Median (min, max) | 1.3 (0.6)  1 (1, 2) | 1.0 (0.0)  1 (1, 1) | 1.2 (0.4)  1 (1, 2) |
| *Contact with a nurse, n (%)* | 7 (35.0) | 10 (41.7) | 17 (38.6) |
| Total number of contacts, Mean (SD)  Median (min, max) | 2.3 (2.0)  1 (1, 6) | 1.8 (1.1)  1 (1, 4) | 2.0 (1.5)  1 (1, 6) |
| Number at surgery, Mean (SD)  Median (min, max) | 1.1 (1.5)  1 (0, 4) | 1.3 (1.1)  1 (0, 3) | 1.2 (1.2)  1 (0, 4) |
| Number at home, Mean (SD)  Median (min, max) | 1.1 (2.2)  0 (0, 6) | 0.4 (1.3)  0 (0, 4) | 0.7 (1.7)  0 (0, 6) |
| Number via telephone, Mean (SD)  Median (min, max) | 0.0 (0.0)  0 (0, 0) | 0.0 (0.0)  0 (0, 0) | 0.0 (0.0)  0 (0, 0) |

Supplementary Table 6b: EQ-5D-5L, and health service use during previous 4 weeks, at weeks 8 and 12 by randomised group

| **EQ-5D-5L, and health service use during previous 4 weeks** | **Morphine** | **Placebo** | **Total** |
| --- | --- | --- | --- |
| ***Week 8*** | **N=20** | **N=23** | **N=43** |
| EQ-5D-5L index value, Mean (SD)  Median (min, max) | 0.68 (0.20)  0.72 (0.24, 1.00) | 0.58 (0.34)  0.66 (-0.51, 1.00) | 0.63 (0.29)  0.68 (-0.51, 1.00) |
| EQ-5D-5L VAS, Mean (SD)  Median (min, max) | 56.5 (20.4)  52.5 (10, 95) | 54.3 (21.0)  60 (0, 90) | 55.3 (20.5)  55 (0, 95) |
| *Overnight stays in hospital, n (%)* | 1 (5.0) | 5 (21.7) | 6 (14.0) |
| Total number of nights, Mean (SD)  Median (min, max) | 11.0 (-)  11 (11, 11) | 10.0 (10.3)  7 (1, 26) | 10.2 (9.2)  9 (1, 26) |
| *Outpatient appointment, n (%)* | 5 (25.0) | 7 (30.4) | 12 (27.9) |
| Total number of visits, Mean (SD)  Median (min, max) | 1.2 (0.4)  1 (1, 2) | 1.9 (1.1)  2 (1, 4) | 1.6 (0.9)  1 (1, 4) |
| *Contact with GP, n (%)* | 8 (40.0) | 8 (34.8) | 16 (37.2) |
| Total number of contacts, Mean (SD)  Median (min, max) | 1.8 (0.9)  1.5 (1, 3) | 1.4 (0.7)  1 (1, 3) | 1.6 (0.8)  1 (1, 3) |
| Number at surgery, Mean (SD)  Median (min, max) | 1.3 (0.7)  1 (0, 2) | 1.3 (0.9)  1 (0, 3) | 1.3 (0.8)  1 (0, 3) |
| Number at home, Mean (SD)  Median (min, max) | 0.0 (0.0)  0 (0, 0) | 0.1 (0.4)  0 (0, 1) | 0.1 (0.3)  0 (0, 1) |
| Number via telephone, Mean (SD)  Median (min, max) | 0.5 (1.1)  0 (0, 3) | 0.0 (0.0)  0 (0, 0) | 0.3 (0.8)  0 (0, 3) |
| *Treatment at A&E department, n (%)* | 1 (5.0) | 3 (13.0) | 4 (9.3) |
| Total number of visits, Mean (SD)  Median (min, max) | 1.0 (-)  1 (1, 1) | 1.0 (0.0)  1 (1, 1) | 1.0 (0.0)  1 (1, 1) |
| *Contact with a nurse, n (%)* | 6 (30.0) | 7 (30.4) | 13 (30.2) |
| Total number of contacts, Mean (SD)  Median (min, max) | 3.2 (1.2)  3 (2, 5) | 1.3 (0.5)  1 (1, 2) | 2.2 (1.3)  2 (1, 5) |
| Number at surgery, Mean (SD)  Median (min, max) | 1.7 (0.8)  1.5 (1, 3) | 0.9 (0.7)  1 (0, 2) | 1.2 (0.8)  1 (0, 3) |
| Number at home, Mean (SD)  Median (min, max) | 0.8 (1.0)  0.5 (0, 2) | 0.3 (0.5)  0 (0, 1) | 0.6 (0.8)  0 (0, 2) |
| Number via telephone, Mean (SD)  Median (min, max) | 0.7 (1.0)  0 (0, 2) | 0.2 (0.4)  0 (0, 1) | 0.4 (0.8)  0 (0, 2) |
| ***Week 12*** | **N=20** | **N=22** | **N=42** |
| EQ-5D-5L index value, Mean (SD)  Median (min, max) | 0.58 (0.21)  0.63 (0.04, 0.88) | 0.66 (0.17)  0.67 (0.08, 0.84) | 0.62 (0.19)  0.66 (0.04, 0.88) |
| EQ-5D-5L VAS, Mean (SD)  Median (min, max) | 55.9 (18.6)  50 (10, 92) | 59.8 (15.6)  60 (25, 90) | 57.9 (17.0)  60 (10, 92) |
| *Overnight stays in hospital, n (%)* | 3 (15.0) | 3 (13.6) | 6 (14.3) |
| Total number of nights, Mean (SD)  Median (min, max) | 12.0 (6.1)  15 (5, 16) | 4.0 (3.0)  4 (1, 7) | 8.0 (6.1)  6 (1, 16) |
| *Outpatient appointment, n (%)* | 12 (60.0) | 6 (27.3) | 18 (42.9) |
| Total number of visits, Mean (SD)  Median (min, max) | 1.6 (1.1)  1 (1, 4) | 1.2 (0.4)  1 (1, 2) | 1.4 (0.9)  1 (1, 4) |
| *Contact with GP, n (%)* | 14 (70.0) | 6 (27.3) | 20 (47.6) |
| Total number of contacts, Mean (SD)  Median (min, max) | 1.4 (0.9)  1 (1, 4) | 1.7 (0.5)  2 (1, 2) | 1.5 (0.9)  1 (1, 4) |
| Number at surgery, Mean (SD)  Median (min, max) | 1.1 (0.8)  1 (0, 3) | 1.5 (0.5)  1.5 (1, 2) | 1.3 (0.7)  1 (0, 3) |
| Number at home, Mean (SD)  Median (min, max) | 0.1 (0.3)  0 (0, 1) | 0.0 (0.0)  0 (0, 0) | 0.1 (0.2)  0 (0, 1) |
| Number via telephone, Mean (SD)  Median (min, max) | 0.4 (0.7)  0 (0, 2) | 0.2 (0.4)  0 (0, 1) | 0.3 (0.6)  0 (0, 2) |
| *Treatment at A&E department, n (%)* | 4 (20.0) | 4 (18.2) | 8 (19.1) |
| Total number of visits, Mean (SD)  Median (min, max) | 1.0 (0.0)  1 (1, 1) | 1.0 (0.0)  1 (1, 1) | 1.0 (0.0)  1 (1, 1) |
| *Contact with a nurse, n (%)* | 5 (25.0) | 9 (40.9) | 14 (33.3) |
| Total number of contacts, Mean (SD)  Median (min, max) | 1.8 (0.8)  2.0 (1, 3) | 2.1 (2.3)  1.0 (1, 8) | 2.0 (1.9)  1 (1, 8) |
| Number at surgery, Mean (SD)  Median (min, max) | 0.8 (0.4)  1 (0, 1) | 0.8 (0.7)  1 (0, 2) | 0.8 (0.6)  1 (0, 2) |
| Number at home, Mean (SD)  Median (min, max) | 0.8 (1.0)  0.5 (0, 2) | 1.1 (2.6)  0 (0, 8) | 1.0 (2.2)  0 (0, 8) |
| Number via telephone, Mean (SD)  Median (min, max) | 0.0 (0.0)  0 (0, 0) | 0.2 (0.7)  0 (0, 2) | 0.2 (0.6)  0 (0, 2) |

**Supplementary Figure 1.**
